# Analysis of Pain Management after Anatomic VATS Resection in Austrian Thoracic Surgery Units

**DOI:** 10.3390/jcm13010080

**Published:** 2023-12-22

**Authors:** Florian Ponholzer, Thomas Schweiger, Bahil Ghanim, Herbert Maier, Jörg Hutter, Florian Tomaselli, Axel Krause, Michael Müller, Jörg Lindenmann, Gero Spruk, Florian Augustin

**Affiliations:** 1 Department of Visceral, Transplant and Thoracic Surgery, Center of Operative Medicine, Medical University of Innsbruck, 6020 Innsbruck, Austria; florian.ponholzer@i-med.ac.at (F.P.); herbert.maier@tirol-kliniken.at (H.M.); 2 Division of Thoracic Surgery, Department of Surgery, Medical University of Vienna, 1090 Vienna, Austria; 3 Department of General and Thoracic Surgery, University Hospital Krems, Karl Landsteiner University of Health Sciences, 3500 Krems an der Donau, Austria; bahil.ghanim@krems.lknoe.at; 4 Department of Surgery, Paracelsus Medical University, 5020 Salzburg, Austria; j.hutter@aon.at; 5 Department of Cardiac-, Vascular-, and Thoracic Surgery, Johannes Kepler University Linz, 4020 Linz, Austria; 6 Department of Surgery, Elisabethinen Hospital, 4020 Linz, Austria; 7 Department of Thoracic Surgery, Clinic Floridsdorf, 1210 Vienna, Austria; michael.mueller@gesundheitsverbund.at; 8 Division of Thoracic and Hyperbaric Surgery, Department of Surgery, Medical University of Graz, 8010 Graz, Austria; 9 Department of Cardiac-, Thoracic- and Vascular Surgery, Klinikum Klagenfurt am Wörthersee, 9020 Klagenfurt, Austria; gero.spruk@kabeg.at

**Keywords:** VATS, pain management, opioid, postoperative pain, multicenter

## Abstract

Background: Postoperative pain influences rehabilitation, postoperative complications and quality of life. Despite its impact, there are no uniform treatment guidelines. Different centers seem to use various strategies. This study aims to analyze pain management regimens used after anatomic VATS resections in Austrian thoracic surgery units, with a special interest in opioid usage and strategies to avoid opioids. Methods: A questionnaire was designed to assess the use of regional anesthesia, postoperative pain medication and characteristics of individual pain management regimens. The questionnaire was sent to all thoracic surgery units in Austria, with nine out of twelve departments returning them. Results: All departments use regional anesthesia during the procedure. Four out of nine centers use epidural analgesia or an intercostal catheter for postoperative regional anesthesia in at least 50% of patients. Two departments follow an opioid restrictive regimen, five depend on the visual analogue scale (VAS) and two administer opioids on a fixed schedule. Three out of nine departments use NSAIDs on a fixed schedule. The most used medication is metamizole (eight out of nine centers; six on a fixed schedule, two depending on VAS) followed by piritramide (six out of nine centers; none as a fixed prescription). Conclusions: This study reflects the heterogeneity in postoperative pain treatment after VATS anatomic lung resections. All departments use some form of regional anesthesia in the perioperative period; prolonged regional anesthesia is not utilized uniformly to reduce opioid consumption, as suggested in enhanced recovery after surgery programs. More evidence is needed to optimize and standardize postoperative pain treatment.

## 1. Introduction

Postoperative pain in thoracic surgery influences recovery, promotes pulmonary complications and impacts quality of life. Despite its importance, postoperative pain management after video-assisted thoracoscopic surgery (VATS) resection is still a subject of discussion lacking evidence-based guidelines. Many departments use their own pain treatment regimens with various modalities for postoperative analgesia. Rationale for these regimens is based on various sources including the available literature and in-house standards resulting from interprofessional discussions, but also personal opinions reflecting an eminence-based approach to this topic. As a result, pain management regimes may not be tailored to the needs of VATS patients but are rather taken from historical thoracotomy protocols or other specialties’ standards, leading to possibly reduced therapy success. The available literature is lacking concordant results and recommendations with an overall low level of evidence [1,2,3,4]. Guidelines from the Enhanced Recovery After Surgery (ERAS) Society and the European Society of Thoracic Surgeons (ESTS) provide general recommendations regarding management of patients after lung surgery. These guidelines recommend a primary minimally invasive approach to improve postoperative outcomes. A multimodal pain management regimen with regional anesthesia is recommended to reduce the need for postoperative opioids. Regarding regional analgesia, the ERAS/ESTS guidelines prefer a paravertebral block over thoracic epidural analgesia due to reduced side effects and no associated differences in acute pain levels [5]. However, despite the efforts put into these recommendations, specific guidelines for optimal pain management of VATS patients are missing.

In general, postoperative pain is a severe issue across many surgical specialties. Even with the available pain management regimens, postoperative pain often leads to chronic postsurgical pain, inflicting not only budgetary pressure on public health care providers, but also impacting patients’ quality of life. One year after any type of surgery, 11.8% of European patients still experience moderate to severe pain linked to their surgery. Functional impairment seems to increase the severity of postoperative pain and, thus, calling for pain management regimens, which not only ease patients´ pain, but also offer fast rehabilitation [6,7]. Chronic pain might also constrain patients from returning to their daily life and routine, which poses a hazard to mental health and satisfaction, while also involving a loss of income for patients and their employees. Subsequently, these patients require further medical follow-up and therapies, which not only raises health care costs, but also blocks valuable treatment spots. Appropriate pain management regimes in the first place might be able to avoid these sequelae.

Opioids remain an important medication in many pain management regimens, although a trend towards reducing opioid consumption can be seen [8,9]. This trend is especially important in the field of thoracic surgery as the most common adverse effects of opioids, like respiratory depression, can directly impact or lead to postoperative complications and possibly delay further needed treatment [8,9,10,11]. The raging opioid epidemic in the United States, which is leading to a rising number of overdose deaths, calls for further optimization of postoperative pain management. Opioid use disorders tend to start with prescription opioid use in combination with other prescribed drugs, such as benzodiazepines and psychostimulants. Therefore, it is of utmost importance to already prevent a need for opioids by adequately treating postoperative pain with standardized multimodal pain management regimes [12].

The aim of our study was to investigate the different pain treatment modalities in Austrian thoracic surgery departments. Due to its recent importance and widespread use, we put special focus on opioid usage within the different regimens of postoperative pain treatment.

Modern analgesia follows a multidisciplinary approach and includes regional anesthesia techniques, which are performed more and more by surgeons. As an example, we regularly place an intercostal catheter for the application of local anesthetics. A secondary objective of this study is to evaluate the feasibility of a possible prospective, randomized controlled trial in Austrian thoracic departments to analyze the impact of regional anesthesia (RA) for improved pain management.

## 2. Materials and Methods

For this study, a self-designed questionnaire with nine questions was used and sent to all twelve thoracic surgery units in Austria. Questions focused on the use of RA, postoperative pain medication, individual pain management regimens and the basis of these. The questionnaire was created to collect data on current pain management regimens without asking for recommendations or individual opinions. Questions were designed by three authors of this study. Questions were accepted if the authors were unanimous on their value and phrasing. The survey was carried out in German and questions were discussed with participants to avoid possible misinterpretations. An overview of the questionnaire and the received answers can be found in Table 1.

If there was no response after two weeks, a reminder was sent. Questions could have multiple answers and free-text answers. Unclear answers on the questionnaire were enquired via personal contact. 

The returned questionnaires were analyzed and transferred to an Excel sheet. Questionnaires not received after a reminder were excluded from analysis.

Approval of our institutions ethics committees was not necessary, as no patient data were involved.

## 3. Results

The overall response rate was nine out of twelve departments (75%). All departments answered each question and left no missing data.

The number of performed minimally invasive anatomical resections ranged from 32 to 250 per year (median: 100/year, mean: 114.0/year) between centers. Regarding the surgical approach, one out of nine (11.1%) centers uses a uniportal approach, while the other eight (88.9%) prefer a multiportal (two or more ports) approach. Seven (77.8%) out of nine centers use a digital thoracic drainage system after anatomical VATS resection and two (22.2%) used a conventional water seal system. Criteria for the removal of chest drains also varied between centers. The allowed drainage amount for chest drain removal was between 300 mL/24 h and 400 mL/24 h (without signs of active bleeding). One center uses a weight-adapted calculation of allowed drainage amount in mL/24 h: 4.8 × body weight in kilograms. The allowed air flow before chest drain removal ranged from 0 mL/min to <40 mL/min. Centers using conventional water seal systems required no visual detection of air bubbles during coughs.

**Figure 1:**
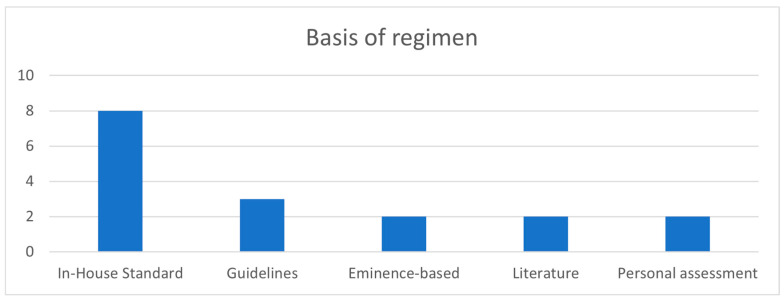
Overview of the basis of the different regimens used for pain management.

All departments reported to use a standardized pain management regimen for all their VATS patients. Asked about the basis of their regimens, eight departments (88.9%) state that it is based on an in-house standard. Three (33.3%) departments additionally refer to available guidelines and two (22.2%) to the published literature. Two (22.2%) participating centers build their treatment approach on eminence-based opinion. Apart from the in-house standard, two (22.2%) centers adjust pain management based on the personal assessment of patients. An overview of the different strategies can be seen in Figure 1. No department used the same regimen.

All centers use a visual analog scale or numeric rating scale to document their patients´ pain scores daily.

### 3.1. Regional Anesthesia

All departments use regional anesthesia as part of the postoperative pain management. Techniques used include single-shot strategies like intercostal nerve block (SSINB), thoracic paravertebral block (TPVB), serratus plane block (SPB) and erector spinae plane block (ESPB) in single-shot technique, but also catheter-based techniques like epidural anesthesia (EA) or an intercostal catheter (ICC), offering the possibility to prolong RA. Five out of nine centers (55.6%) use a SSINB in at least 50% of their patients, three an ICC (33.3%), two an EA (22.2%), one a TPVB (11.1%) in single-shot technique, or a combination of these modalities. Serratus plane block is not used by any of the participating centers on a regular basis. An overview is visualized in Table 2.

When using some form of catheterized postoperative regional anesthesia, six out of nine centers (66.7%) use a continuous form of application in combination with patient-controlled analgesia (PCA).

While a single-shot intercostal nerve block is always performed by the thoracic surgeon, other regional anesthesia modalities are performed by both thoracic surgeons and anesthesiologists.

### 3.2. Opioid Usage

Only two centers (22.2%) reported to use an opioid restrictive and, if possible, an opioid-free approach. Five units (55.6%) administer opioids based on patient demand if a certain threshold in the visual analog scale for pain (VAS) is reached. Two centers (22.2%) administer opioids on a fixed schedule, independently of VAS, in the early postoperative period. The most used opioid was piritramide (six centers), with two centers using it intravenously, two using it solely subcutaneously and two using both types of application. No department prescribes opioids as discharge medication, if possible.

### 3.3. Postoperative Pain Medication

The most used postoperative pain medication was metamizole, with eight departments (88.9%) using it in their in-house standard (six on a fixed schedule and two depending on VAS), followed by piritramide, which is used by six departments (66.7%; all six depending on VAS), and paracetamol by five departments (55.6%; four on a fixed schedule and one depending on VAS). The most optional medication was diclofenac, with three centers (33.3%) using it standardly and another three using it as a reserve medication. Table 3 shows an overview of the most used medications.

### 3.4. Discharge Medication

The most widely used discharge medication is metamizole, being prescribed in five centers (55.6%), followed by NSAIDs (five centers; 55.6%) and paracetamol (three centers; 33.3%).

## 4. Discussion

Pain after thoracic surgery remains a clinically relevant problem. It hampers early mobilization and mucus clearance, thus provoking potentially fatal pulmonary complications. While acute postsurgical pain diminishes over time, chronic pain can largely influence rehabilitation, return to daily activities and quality of life. Due to its importance, it is surprising that there is only little evidence in postoperative pain management after minimally invasive lung resections, with no guidelines available to date [1,2,3,4]. Although patients after a VATS resection—in comparison to thoracotomy—benefit from a minimally invasive approach, they are not pain free [13]. Even the available ERAS/ESTS guidelines for enhanced recovery after lung surgery have no dedicated section on pain management regimens for VATS patients only. Although minimally invasive surgical approaches are recommended, no clear distinction is made to open surgery regarding pain management recommendations [5]. It seems rational to evaluate existing regimens and to start comparing them to optimize postoperative pain management and the associated morbidity and quality of life to further improve postoperative outcomes.

In order to assess the variability of pain management in Austria, we performed a survey in all Austrian thoracic surgery units. Nine out of twelve centers responded to the questionnaire, thus representing the majority of daily practice in Austria. Every department used its unique regimen of postoperative pain medications. Nevertheless, all departments use some form of regional anesthesia. The route of administration varies from epidural analgesia, which was considered the gold standard in the thoracotomy era, and different peripheral blocks, usually placed by the anesthesiologist under ultrasound guidance (TPVB, ESPB and SPB), to single-shot intercostal injections. Some of these were also used as catheter-based RA to reduce pain for a longer duration. In accordance to ERAS protocols, using regional anesthesia should reduce the amount of opioids used in the postoperative period. The high variability of different regional anesthesia modalities reflects the lack of evidence for postoperative pain management. Moreover, it seems highly likely that the use of epidural analgesia reflects a simple transfer of pain management strategies from the times of open thoracic surgery. This also points to the fact that there is no uniformly accepted optimal pain management for VATS patients.

As can be seen by our data, opioids still play an important role in postoperative analgesia, with only two departments following an opioid-restrictive approach. This seems to be a discrepancy with recent trends to reduce the use of opioids and move to regional anesthesia and other forms of adjuvant therapy [8,9]. An explanation for this might be the cheap and wide availability of opioids in comparison to the sometimes extensive amounts of work and resources needed to implement new treatment modalities, especially in the area of regional anesthesia. This could be simplified by using already available infrastructure, knowledge and materials. ICCs represent such an alternative, as they use the same equipment as an EA, are easy to perform and are a feasible option to reduce opioid usage [14]. Moreover, ICCs do not add significant delay to operating time, which might further improve its acceptance as an addition to already-available pain management systems [15]. As pain after VATS resections seems to originate from the area of the chest drain and is in decline after chest drain removal, ICCs provide a feasible option to overcome this initially painful phase by locally targeting the affected intercostal space [15,16]. Therefore, we also evaluated the chest drain management of participating centers. Seven out of nine routinely use a digital thoracic drainage system, while the other two use conventional water seal systems. Criteria for removal of the chest drain also varied between centers with the allowed drainage amount ranging from 300 mL/24 h to 400 mL/24 h. Also, the accepted air flow at time of chest drain removal ranged from 0 mL/min to <40 mL/min. A subsequent reduction in postoperative opioid consumption might even lower postoperative complications, as opioids have a depressant effect on the respiratory system and might lead to atelectasis and pneumonia as a consequence. This effect might be amplified further by the common use of short-acting opioids by anesthesiologists in the post-anesthesia care unit due to prolonged infusion in the operating theatre. Moreover, if patients also require sedatives, their effects with opioids can be of a synergistic nature, leading to further impairment of the patient’s wakefulness and hypoxic and hypercapnic chemoreflexes [10,11]. This combination of cumbersome effects may prolong a patient’s time on the post-anesthesia care unit and hinder early mobilization. Through this contradiction to existing ERAS protocols, recovery time is slowed and morbidity elevated. Unfortunately, surgeons often have little to no influence on pain management in the first hours following surgery, as anesthesiologists, understandably, remain in charge. The only way for surgeons to influence this period is through perioperative interventions, such as placement of an ICC to reduce the opioid demand of patients.

Currently, there is no standardized postoperative pain management regime after VATS, which explains the heterogeneous answers of the participating centers in this study. Although all returned questionnaires showed different regimens, it was also apparent that all departments use some form of regional anesthesia and that six use a continuous form of application in combination with PCA postoperatively. Also, postoperative intravenous, oral and subcutaneous medication was similar between centers. Considering this variability and the various different bases of the used regimens, a universal pain management guideline should be implemented and subsequently analyzed. This publication might be used as a basis to find similarities between centers and form a mutually agreed consensus.

Even though metamizole is banned in many countries, including the United States, United Kingdom and various countries in the European Union, it remains the most used postoperative medication in Austrian thoracic surgery departments. This is in accordance with recent meta-analyses and reviews, which show that metamizole is a safe analgesic agent for short periods of treatment and that the number of adverse events does not differ from placebo, paracetamol or other NSAIDs. Nevertheless, metamizole shows less reported adverse events than opioids, which emphasizes the advantage of its use [17,18]. The ban of metamizole in some countries is linked to the potential risk of drug-induced agranulocytosis, which has been associated with specific HLA alleles. This might be an explanation for the highly varying reported incidence of metamizole-induced agranulocytosis [19,20]. Considering this, future guidelines might have to be adaptable to specific regions or ethnicities and provide different pain management strategies to cover a wider range of regions and patients. If proposed regimes do not acknowledge these regional differences, it might be difficult to reach a consensus.

Postoperative pain management also influences patients’ quality of life and their rehabilitation, placing not only a strain on the public health care sector, but also impacting the non-public economy. Therefore, it is important to intercept postoperative pain before chronic manifestation or constant opioid usage sets in [21,22]. Postoperative chronic pain is a common complication after thoracic surgery, including minimally invasive VATS. Studies report incidences from 7.7% to up to 47% of patients suffering from postoperative chronic pain. Nevertheless, chronic pain after VATS is significantly reduced in comparison to thoracotomy [23,24,25]. Patients with clinically significant chronic pain mostly localize it at the area of the scar (89.6%) or in the scar (26.1%), with more than half of the affected study cohort describing aching or numbness. Interestingly, chronic pain patients experienced significantly more pain when coughing in comparison to VATS patients without chronic pain [26]. This pain development might be due to cortical reorganization after surgery. Cortical reorganization can be triggered through damaged nerves continuing to send signals or damaged inhibitory C-fiber nerves. Through the loss of inhibitory C-fiber nerves, prior inhibited nerves may be activated and send pain signals to the cortex [27]. This initial development of chronic pain might be disrupted by the intraoperative placement of postoperative regional anesthesia, such as ICCs, which have already successfully proven to significantly reduce postoperative pain and opioid consumption in VATS patients [15]. Nevertheless, data regarding its influence on the development of chronic pain are still lacking and needs to be investigated in further follow-up studies.

Our investigation can be seen as a feasibility study for a further workup to optimize postoperative pain management and analyze the rising role of regional anesthesia and its influence not only on acute postoperative pain, but also on the development of postoperative chronic pain. The advancing introduction of lung cancer screening programs is leading to higher numbers of diagnoses of early-stage lung cancers [28]. These often represent candidates for surgical treatment as first-line therapy according to recent data and guidelines [29]. This will result in higher rates of surgically treated patients and naturally higher absolute numbers of patients with postoperative chronic pain syndromes. To reduce the socio-economic effect of this inevitable development, measures to optimize postoperative pain management have to be taken. Without standardization and comparability of pain therapy strategies, no significant progress will be achieved. Furthermore, the management of chronic pain patients requires additional medical personnel, who are already scarce in current times. As all of the departments demonstrate the needed infrastructure and experience at hand, it is possible to initiate a prospective study to compare postoperative regional anesthesia with intraoperative regional anesthesia alone and its impact on patients’ experienced pain, duration of rehabilitation, chronic pain development and quality of life. When conducting prospective studies, secondary endpoints such as return to daily life, return to work and physical capacity have to be considered too. Otherwise, the efficacy of pain management regimes regarding above-mentioned factors cannot be correlated with their socio-economic effects. 

These efforts might be the cornerstone for future guidelines regarding pain management for patients receiving (minimally invasive) thoracic surgery.

## 5. Limitations

Each questionnaire was completed by one team member and might not always reflect the everyday pain management regimens of other employees following their personal opinion. Pain management remains a complex topic, being also heavily influenced by patients’ subjective experience of pain. Existing comorbidities might further influence individual pain management regimes. Due to the retrospective nature of this study, no data regarding the days until removal of the chest drain and its impact on pain (management) are available. Although questions have been discussed with the participating centers, misinterpretations of questions are possible.

## 6. Conclusions

Postoperative pain management varies between departments, but revolves around the same modalities, including regional anesthesia. Thoracic surgeons together with their anesthesiologists need to focus on optimizing postoperative pain management, ideally in the setting of randomized controlled trials.

## Figures and Tables

**Table 1:** Overview of the questionnaire.

Do You Use Regional Anesthesia Procedures as a Standard for VATS Lobectomies?	Yes: 9, No: 0
- Intercostal nerve block (ICNB; single-shot intraoperative)	5
- Peridural/epidural anesthesia (PDA)	2
- Intercostal catheter (ICC)	3
- Thoracic paravertebral block (TPVB)	1
- Serratus plane block	0
- Others:	0
If regional anesthesia procedures are used, is a continuous flow rate or patient-controlled analgesia (PCA) used?	
- Continuous	0
- PCA	0
- Continuous with patient-controlled bolus delivery	6
Does your department have a standard for pain management in patients after VATS lobectomies?	
- Yes	9
- No	0
Which of the following pain medications do your patients receive as standard therapy during their inpatient stay after VATS lobectomy? If medication is administered at VAS, please specify from which VAS range.	
- Paracetamol	5/9
- Fixed schedule or at VAS	4 vs. 1
- Mode of administration: oral intravenous	both in 5/5
- Ibuprofen	1/9
- Fixed schedule or at VAS	1 vs. 0
- Mode of administration: oral intravenous	oral
- Dexibuprofen	0/9
- Fixed schedule or at VAS	n.a.
- Metamizole	8/9
- Fixed schedule or at VAS	6 vs. 2
- Mode of administration: oral intravenous	both in 8/8
- Diclofenac	3/9
- Fixed schedule or at VAS	2 vs. 1
- Mode of administration: oral intravenous	both in 3/3
- Gabapentin	0/9
- Fixed schedule or at VAS	n.a.
- Mode of administration: oral intravenous	n.a.
- Piritramide	6/9
- Fixed schedule or at VAS	0 vs. 6
- Mode of administration: intravenous subcutaneous	both in 6/6
- Hydromorphone hydrochloride	4/9
- Fixed schedule or at VAS	1 vs. 3
- Mode of administration: oral intravenous subcutaneous	oral in 4/4
- Tramadol hydrochloride	1/9
- Fixed schedule or at VAS	0 vs. 1
- Mode of administration: oral intravenous subcutaneous	oral in 1/1
- Others: - Lornoxicam, Oxycodon, Oxycodon/Naloxon, Diclofenac/Orphenadrin	one each, 3 on a fixed schedule, Oxycodon depending on VAS
How restrictively are opiates used for postoperative pain management in your practice?	
- Opioids are prescribed for every patient	2/9
- According to VAS. If so, from which VAS:	5/9
- Rather restrictive/attempt opiate-free analgesia	2/9
Which of the following is your pain medication regimen based on? (multiple answers possible)	
- Guidelines	3
- Literature	2
- “Eminence-based”	2
- In-house standard	8
- Personal assessment	2
- Other:	0
What pain medication do your patients receive at discharge and for how long?	Metamizole in 5/9, NSAID in 5/9, Paracetamol in 3/9, Duration based on symptoms in 9/9
Do you use any other procedures for analgesia during or after the patient’s inpatient stay, which have not yet been explained in the above questions?	No: 9/9
Are your patients regularly surveyed regarding their pain?	
- No	0
- Yes. If so: at what times?	9/9, median 3 times/24 h, range 3–6 times/24 h
How many anatomical minimally invasive resections are performed at your center per year?	median 100/y, range 32–250/y
Do you use a uniportal or multiportal (two or more ports) approach?	multiport approach *n* = 8, uniportal *n* = 1
Do you use a digital thoracic drainage system?	Yes: 7, No: 2
Please describe your chest drain management with regard to criteria for drain removal:	
- Do you look at the quality of the fluid?	Yes: 9
- Maximum drainage amount over 24 h?	median 300 mL/24 h, range 100–400 mL/24 h
- If a digital thoracic drainage system is used:	
○ Maximum air flow in mL/min?	median 20 mL/min, range 0–40 mL/min

n.a.: not applicable.

**Table 2:** Overview of used techniques and performing specialty.

Technique	Usage (%)	By Surgeons	By Anesthesiologists
SSINB	5 out of 9 (55.6)	X	
ICC	3 out of 9 (33.3)	X	
EA	2 out of 9 (22.2)		X
TPVB	1 out of 9 (11.1)	X	X

EA: epidural anesthesia, ICC: intercostal catheter, SSINB: single-shot intercostal nerve block, TPVB: thoracic paravertebral block. X indicates the performing specialty.

**Table 3:** Overview of the most used medications.

Medication	Fixed Schedule	Depending on VAS	Overall Usage
Metamizole	6	2	8 out of 9
Piritramide	0	6	6 out of 9
Paracetamol	4	1	5 out of 9
Hydromorphone	1	3	4 out of 9
Diclofenac *	2	1	3 out of 9
Ibuprofen	1	0	1 out of 9
Tramadol	0	1	1 out of 9
Lornoxicam	1	0	1 out of 9
Oxycodon	0	1	1 out of 9
Oxycodon/Naloxon	1	0	1 out of 9
Diclofenac/Orphenadrin	1	0	1 out of 9

* another three centers use diclofenac as reserve medication.

## Data Availability

Data is contained within the article.

## References

[1] Goto T. (2018). What is the best pain control after thoracic surgery?. J. Thorac. Dis..

[2] Mercieri M., D’andrilli A., Arcioni R. (2018). Improving postoperative pain management after video-assisted thoracic surgery lung resection contributes to enhanced recovery, but guidelines are still lacking. J. Thorac. Dis..

[3] Elmore B., Nguyen V., Blank R., Yount K., Lau C. (2015). Pain Management Following Thoracic Surgery. Thorac. Surg. Clin..

[4] Allain P.-A., Carella M., Agrafiotis A.C., Burey J., Assouad J., Hafiani E.-M., Ynineb Y., Bonnet F., Garnier M., Quesnel C. (2019). Comparison of several methods for pain management after video-assisted thoracic surgery for pneumothorax: An observational study. BMC Anesthesiol..

[5] Batchelor T.J.P., Rasburn N.J., Abdelnour-Berchtold E., Brunelli A., Cerfolio R.J., Gonzalez M., Ljungqvist O., Petersen R.H., Popescu W.M., Slinger P.D. (2019). Guidelines for enhanced recovery after lung surgery: Recommendations of the Enhanced Recovery after Surgery (ERAS^®^) Society and the European Society of Thoracic Surgeons (ESTS). Eur. J. Cardio-Thorac. Surg..

[6] Fletcher D., Stamer U.M., Pogatzki-Zahn E., Zaslansky R., Tanase N.V., Perruchoud C., Kranke P., Komann M., Lehman T., Meissner W. (2015). Chronic postsurgical pain in Europe: An observational study. Eur. J. Anaesthesiol..

[7] Guimaraes-Pereira L., Valdoleiros I., Reis P., Abelha F. (2016). Evaluating Persistent Postoperative Pain in One Tertiary Hospital: Incidence, Quality of Life, Associated Factors, and Treatment. Anesthesiol. Pain Med..

[8] Chou R., Gordon D.B., de Leon-Casasola O.A., Rosenberg J.M., Bickler S., Brennan T., Carter T., Cassidy C.L., Chittenden E.H., Degenhardt E. (2016). Management of Postoperative Pain: A Clinical Practice Guideline from the American Pain Society, the American Society of Regional Anesthesia and Pain Medicine, and the American Society of Anesthesiologists’ Committee on Regional Anesthesia, Executive Committee, and Administrative Council. J. Pain.

[9] Thompson C., French D.G., Costache I. (2018). Pain management within an enhanced recovery program after thoracic surgery. J. Thorac. Dis..

[10] Ziarnik E., Grogan E.L. (2015). Post-lobectomy early complications. Thorac. Surg. Clin..

[11] Karcz M., Papadakos P.J. (2013). Respiratory complications in the postanesthesia care unit: A review of pathophysiological mechanisms. Can. J. Respir. Ther..

[12] Blanco C., Wiley T.R.A., Lloyd J.J., Lopez M.F., Volkow N.D. (2020). America’s opioid crisis: The need for an integrated public health approach. Transl. Psychiatry.

[13] Bendixen M., Jørgensen O.D., Kronborg C., Andersen C., Licht P.B. (2016). Postoperative pain and quality of life after lobectomy via video-assisted thoracoscopic surgery or anterolateral thoracotomy for early stage lung cancer: A randomised controlled trial. Lancet Oncol..

[14] Hsieh M.-J., Wang K.-C., Liu H.-P., Gonzalez-Rivas D., Wu C.-Y., Liu Y.-H., Wu Y.-C., Chao Y.-K., Wu C.-F. (2016). Management of acute postoperative pain with continuous intercostal nerve block after single port video-assisted thoracoscopic anatomic resection. J. Thorac. Dis..

[15] Ponholzer F., Ng C., Maier H., Dejaco H., Schlager A., Lucciarini P., Öfner D., Augustin F. (2021). Intercostal Catheters for Postoperative Pain Management in VATS Reduce Opioid Consumption. J. Clin. Med..

[16] Wildgaard K., Petersen R.H., Hansen H.J., Møller-Sørensen H., Ringsted T.K., Kehlet H. (2012). Multimodal analgesic treatment in video-assisted thoracic surgery lobectomy using an intraoperative intercostal catheter. Eur. J. Cardio-Thorac. Surg..

[17] Andrade S., Bartels D.B., Lange R., Sandford L., Gurwitz J. (2016). Safety of metamizole: A systematic review of the literature. J. Clin. Pharm. Ther..

[18] Kötter T., da Costa B.R., Fässler M., Blozik E., Linde K., Jüni P., Reichenbach S., Scherer M. (2015). Metamizole-Associated Adverse Events: A Systematic Review and Meta-Analysis. PLoS ONE.

[19] Hoffmann F., Bantel C., von Rosen F.T., Jobski K. (2020). Regional Differences in Prescribing Patterns of Metamizole in Germany Based on Data from 70 Million Persons. Int. J. Environ. Res. Public Health.

[20] Shah R.R. (2019). Metamizole (dipyrone)-induced agranulocytosis: Does the risk vary according to ethnicity?. J. Clin. Pharm. Ther..

[21] Gostin L.O., Hodge J.G., Noe S.A. (2017). Reframing the Opioid Epidemic as a National Emergency. JAMA.

[22] Guertin J.R., Pagé M.G., Tarride J.-É., Talbot D., Watt-Watson J., Choinière M. (2018). Just how much does it cost? A cost study of chronic pain following cardiac surgery. J. Pain Res..

[23] Tong Y., Wei P., Wang S., Sun Q., Cui Y., Ning N., Chen S., He X. (2020). Characteristics of Postoperative Pain After VATS and Pain-Related Factors: The Experience in National Cancer Center of China. J. Pain Res..

[24] Bayman E.O., Parekh K.R., Keech J., Selte A., Brennan T.J. (2017). A Prospective Study of Chronic Pain after Thoracic Surgery. Anesthesiology.

[25] Zhang Y., Zhou R., Hou B., Tang S., Hao J., Gu X., Ma Z., Zhang J. (2022). Incidence and risk factors for chronic postsurgical pain following video-assisted thoracoscopic surgery: A retrospective study. BMC Surg..

[26] Wang H., Li S., Liang N., Liu W., Liu H., Liu H. (2017). Postoperative pain experiences in Chinese adult patients after thoracotomy and video-assisted thoracic surgery. J. Clin. Nurs..

[27] Flor H. (2002). Painful memories. Can we train chronic pain patients to ‘forget’ their pain?. EMBO Rep..

[28] Babar L., Modi P., Anjum F. (2023). Lung Cancer Screening.

[29] Indini A., Rijavec E., Bareggi C., Grossi F. (2020). Novel treatment strategies for early-stage lung cancer: The oncologist’s perspective. J. Thorac. Dis..

